# Pathogenic pathways are activated in each major cell type of the glomerulus in the *Cd2ap* mutant mouse model of focal segmental glomerulosclerosis

**DOI:** 10.1186/s12882-015-0063-z

**Published:** 2015-05-13

**Authors:** Eric W. Brunskill, S. Steven Potter

**Affiliations:** Cincinnati Children’s Medical Center, 3333 Burnet Ave., Cincinnati, OH 45229 USA

**Keywords:** *Cd2ap*, Focal segmental glomerulosclerosis, Podocytes, Mesangial cells, Endothelial cells, RNA-seq, Pathogenic pathways

## Abstract

**Background:**

Mutations in several genes expressed in podocytes, including *Cd2ap*, have been associated with focal segmental glomerulosclerosis in humans. Mutant mouse models provide an opportunity to better understand the molecular pathology that drives these diseases.

**Methods:**

In this report we use a battery of transgenic-GFP mice to facilitate the purification of all three major cell types of the glomerulus from *Cd2ap* mutant mice. Both microarrays and RNA-seq were used to characterize the gene expression profiles of the podocytes, mesangial cells and endothelial cells, providing a global dual platform cross-validating dataset.

**Results:**

The mesangial cells showed increased expression of profibrotic factors, including thrombospondin, Tgfb2 and Tgfb3, as well as the angiogenesis factor Vegf. They also showed upregulation of protective genes, including *Aldh1a2*, involved in retinoic acid synthesis and *Decorin*, a Tgfb antagonist. Of interest, the mesangial cells also showed significant expression of *Wt1*, which has generally been considered podocyte specific. The *Cd2ap* mutant podocytes showed upregulation of proteases as well as genes involved in muscle and vasculature development and showed a very strong gene expression signature indicating programmed cell death. Endothelial cells showed increased expression of the leukocyte adhesion associated factors Vcam1 and Sele, as well as Midkine (promoting angiogenesis), endothelin and many genes responsive to cytokines and interferons.

**Conclusions:**

This study provides a comprehensive analysis of the changing properties of the three cell types of the glomerulus in *Cd2ap* mutants, identifying activated and repressed pathways and responsible genes, thereby delivering a deeper molecular understanding of this genetic disease.

**Electronic supplementary material:**

The online version of this article (doi:10.1186/s12882-015-0063-z) contains supplementary material, which is available to authorized users.

## Background

The CD2-associated protein (CD2AP) is a widely expressed adapter protein. In the kidney CD2AP is found in podocytes at the slit diaphragm where it interacts with nephrin and podocin [1, 2]. Mutations in *Cd2ap* have been associated with focal segmental glomerulosclerosis (FSGS) in humans [3–5]. Mice with homozygous mutation of *Cd2ap* develop severe nephrotic syndrome, with mesangial cell proliferation, extracellular matrix deposition, glomerulosclerosis, extensive foot process effacement and die within weeks of birth [1]. Mice with *Cd2ap* haploinsufficiency show mesangial expansion and hypercellularity by 9 months of age [5]. Transgene driven podocyte specific expression of *Cd2ap* can rescue the *Cd2ap* homozygous mutant lethality, showing that the podocyte is the primary site of essential *Cd2ap* function in the kidney [6]. The *Cd2ap* mutant mouse is therefore an excellent model system for the study of podocyte dysfunction driven glomerulosclerosis.

The glomerulus is primarily composed of three cell types, the podocytes, mesangial cells and endothelial cells. While the podocyte is often the primary site of injury, subsequent changes in all three cell types can provide major contribution to glomerular disease progression. Mesangial expansion, through proliferation or hypertrophy, as well as increased extracellular matrix, is a common feature of progressive renal disease, including FSGS. Further, diseased renal endothelial cells have been associated with increased leukocyte recruitment [7] and can undergo *de novo* angiogenesis, producing immature and leaky vessels [8]. It is therefore clear that each of these cell types can contribute to glomerular disease.

In this study we examined the altered gene expression profiles of all three major cell types of the glomerulus in *Cd2ap* mutant mice. We used *MafB-GFP*, *Meis1-GFP* and *Tie2-GFP* transgene reporters to facilitate FACS purification of the podocytes, mesangial cells and endothelial cells, respectively, from the glomeruli of wild type and *Cd2ap*^−/−^ mice. Gene expression patterns were determined by both microarray and RNA-seq, thereby providing a global dual platform cross validating dataset. In the mesangial cells we observed elevated expression of pro-fibrotic growth factors including thrombospondin, Tgfβ2 and Tgfβ3, as well as the angiogenesis factor Vegf. Mutant mesangial cells also showed upregulation of the protective genes *Aldh1a2*, involved in retinoic acid synthesis and decorin, a Tgfbeta antagonist. Surprisingly, the mesangial cells, both wild type and mutant, also showed significant expression of *Wt1*, which has generally been thought to be podocyte specific. The mutant podocytes showed upregulation of proteases as well as genes involved in muscle and vascular development and a very strong gene expression signature indicating programmed cell death. Endothelial cells showed upregulation of Midkine (promoting angiogenesis), the potent vasoconstrictor endothelin, many genes responsive to cytokines and interferons and *Vcam1* and *Sele*, which promote leukocyte adhesion. The results identify key pathogenic and protective pathways activated in each of these cell types as a result of the *Cd2ap* mutation.

## Methods

### Mouse strains

The *Cd2ap* mutant (B6.129X1-*Cd2ap*^tm1Shaw^/J) [9] and *Tie2-GFP* (Tg[*TIE2GFP*]287Sato/J) [10] mice were from Jackson Laboratory. *MafB-GFP*, Tg(*Mafb-EGFP*)FT79Gsat and *Meis1-GFP* Tg(*Meis1-EGFP*)FO156Gsat, were from GENSAT/MMRC (http://www.gensat.org/MMRC_report.jsp).

### Animal ethics

All animal experiments were carried out according to protocols approved by the Cincinnati Children’s Medical Center Institutional Animal Care and Use Committee (protocol title “mouse models of focal segmental glomerulosclerosis”, number 3C04035).

### Cell purification

Mice were sacrificed at 5 weeks of age. Average mutant mouse BUN levels were elevated to 313 ± 67 from wild type levels of 19.8 ± 5.3. Glomeruli were isolated as previously described [11] and then enzymatically dissociated and FACS used to isolate the GFP positive cells, as previously described [12].

### RNA purification and amplification

RNA was purified using Qiagen RNeasy Micro kits and used for single round RIboSpia amplification using the Nugen Ovation Pico V2 system and used for Affymetrix Mouse Gene 1.0 ST array hybridization or Nextera Tagmentation and Illumina HiSeq2500 sequencing, ~60 million reads per sample, single end 50.

### Data analysis

Microarray and RNA-seq data was primarly analyzed with GeneSpring 12.6.1-GX-NGS. A typical RNA-seq workflow included filtering for minimum expression of 3 RPKM in at least one sample, Audic Claverie Test for statistical significant difference (P < 0.05) and fold change screen as described in the Results and Discussion. For microarray the workflow included filtering on expression requiring minimum 100 raw signal, moderated *T*-Test (P < 0.05) and fold change screen as described in the Results and Discussion. Genespring, ToppGene (http://toppgene.cchmc.org/) [13], ToppCluster (http://toppcluster.cchmc.org/) [14] and Cytoscape (http://www.cytoscape.org/) [15] were used for functional analysis and preparation of figures. Data is available at GEO (GSE63272).

### Immunofluorescence validations

Immunofluorescent validations were carried out as previously described^33^. Primary antibodies were all from Santa Cruz Biotechnology (Santa Cruz CA) and secondary antibodies were from Invitrogen (Carlsbad, CA), with all dilutions according to manufacturer recommendations.

## Results and discussion

### Mesangial cells

The mesangial cell was first recognized as a distinct glomerular cell type by Zimmerman in 1933 and morphologically and functionally defined by Farquhar and Palade in 1962 [16]. Mesangial expansion is a hallmark of many glomerulopathies, with increased mesangial cell proliferation and matrix production resulting in altered gomerular basement membrane permeability and blood flow.

To better understand the possible involvement of mesangial cells in the *Cd2ap* mutant mouse model of FSGS we performed gene expression profiling using both microarrays and RNA-seq. Mesangial cells from control and *Cd2ap*^−/−^ mutant mice were isolated using a combination of sieving, to first isolate glomeruli, followed by FACS purification of mesangial cells, using the *Meis1-GFP* transgene, as previously described [17]. The quality of the resulting datasets was confirmed using several metrics. First, we examined the independent biological replicates for reproducibility. Second, we analyzed the data for possible cell type contamination. For example, we inspected the mesangial gene profile for the expression of genes representing podocyte cell markers, finding very low levels. In addition, by performing the profiling with two independent technologies, RNA-seq and microarray, the resulting datasets provided global cross-validation.

**Fig. 1:**
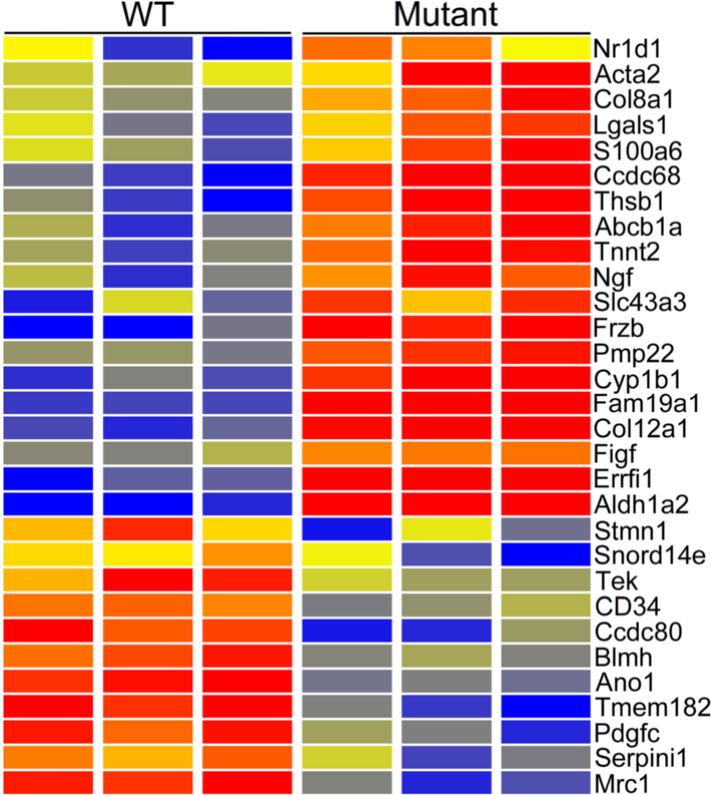
Heatmap of genes showing microarray based differential expression in *Cd2ap* ^−/−^ mutant mesangial cells. Genes were filtered for moderate to high expression (raw signal > 500) and Fold Change > 2. Red is high expression, blue is low expression and yellow is intermediate. Reproducibility is high for the replicates. Differential expression was validated for all of these genes by independent RNA-seq analysis

Analysis of the microarray data identified 176 genes up-regulated (Additional file 1: Table S1) and 265 genes down-regulated (Additional file 2: Table S2) in *Cd2ap* mutant mesangial cells, with P < 0.05 and fold change (FC) > 1.5. Over 90 % of the differences called by microarray were confirmed by independent RNA-seq data analysis (Additional files 1 and 2: Tables S1-S2). A more stringent screen of the array data (raw signal > 500, FC > 2) identified 30 of the most strongly differentially expressed genes (Fig. 1).

The RNA-seq data (RPKM > 3, P < 0.05, FC > 2) identified significantly more differentially expressed genes, with 580 up-regulated (Additional file 3: Table S3) and 420 down-regulated (Additional file 4: Table S4). This RNA-seq/microarray discrepancy is commonly observed and is the result, in part, of the higher background with microarrays, which results in a fold change compression. Perhaps surprising, however, many of the genes with even the greatest fold change, as determined by RNA-seq, were not confirmed by microarray. In most cases this was the result of a failure of the microarray to detect expression of the gene, even though the RNA-seq data looked robust, with high numbers of properly aligned reads for the gene of interest. This could be the result of the microarray target amplification chemistry used and/or array design.

**Fig. 2:**
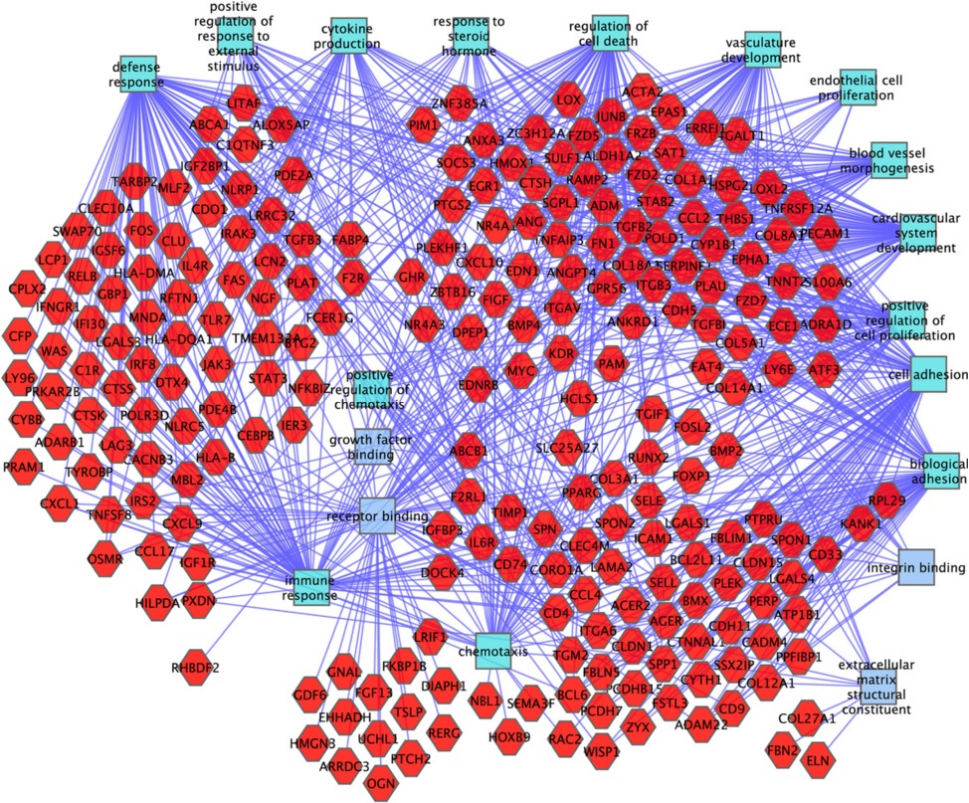
Functional analysis of genes upregulated in mutant mesangial cells. Genes are red hexagons and molecular functions and biological processes are rectangles. Differential expression was defined by RNA-seq. Key functional categories include extracellular matrix component, vasculature development, defense response, regulation of cell death and chemotaxis

Functional enrichment gene ontology analysis identified upregulation in mutants of many genes encoding extracellular matrix components, including Fibrillin2, which can regulate Tgfβ bioavailability, six collagen genes, fibronectin, which is involved in wound healing and collagen deposition in osteoblasts, as well as elastin and CD4, generally associated with immune cells (Additional file 5: Table S5) (Fig. 2).

Also up-regulated in mutant mesangial cells were the Wnt signaling genes *Frzb*, *Fzd2*, *Fzd5* and *Fzd7* and several cytokine/chemokine/growth factors, including the chemokine *Ccl2*, associated with proliferative glomerulonephritis [18], *Ccl4*, associated with idiopathic steroid sensitive nephritic syndrome [19], *Ccl17*, *Cxcl1*, *Cxcl9*, *Cxcl10*, *Bmp2*, *Bmp4* and another BMP family member, *Gdf6* (over 100 fold change), the potent vasoconstrictor *Edn1* (endothelin), *Tnfsf8*, *Tslp*, *Spp1* (nephropontin, with over 100 fold change), *Ngf, Itga6* and *Kdr* (*Vegf*).

Also strongly up-regulated were *Tgfβ2* and *Tgfβ3*, likely of key importance given the strong causal relationship established between Tgfβ and renal fibrosis [20]. Thrombospondin (*Thbs1*) was also dramatically up-regulated, over ten fold, in mutants. Tgfβ is secreted in an inactive pro-cytokine form. Thbs1 plays a key role in Tgfβ activation, with the inflammatory phenotype of *Thbs1* mutants closely resembling that of *Tgfβ* mutants [21]. *Thbs1* is also up-regulated in the mesangial cells of mice with diabetic nephropathy [17].

This powerful cocktail of extracellular matrix and growth factor genes upregulated in the mesangial cells of the *Cd2ap* mutants demonstrates the important role of these cells in disease progression.

One of the most strongly up-regulated genes in the mutant mesangial cells was *Aldh1a2*, which catalyzes the synthesis of retinoic acid. The RNA-seq data showed an up-regulation of 26 fold, with the RPKM going from 13 in wild type to 342 in mutants, indicating very strong expression. The microarray data showed this gene with the greatest fold up-regulation, 8.7. There is an interesting connection between retinoic acid and FSGS. Retinoic acid has been shown to play an important role in the activation of podocyte differentiation genes. In mice with Adriamycin nephropathy, a model of human FSGS, blocking the synthesis of retinoic acid resulted in elevated proteinuria and exacerbated glomerulosclerosis, while treatment with retinoic acid reduced proteinuria and increased podocyte number [22]. The increased *Aldh1a2* expression in *Cd2ap*^−/−^ mutants would therefore be viewed as a protective response, with increased paracrine retinoic acid signaling to nearby podocytes.

It is also interesting to note that there was an approximate three fold increase in *Pparg* expression in mutant mesangial cells, suggesting a possible retinoic acid related autocrine pathway. Pparg forms heterodimers with retinoid X receptors (RXRs) to regulate transcription of target genes. Pparg has been implicated in the pathogenesis of a variety of diseases and plays important roles in regulating proliferation, fibrosis and inflammation. Elevated Pparg in the mutant mesangial cells is likely protective, as agonists of Pparg have been shown to reduce disease progression for renal fibrosis [23], cystogenesis in embryonic *Pkd1*^−/−^ and adult *Pkd1*^+/−^ mice [24] and in *Pck* rats [25].

Another likely protective gene expression change in the mesangial cells of mutants was the strongly elevated expression of Decorin (*Dcn*). Dcn can interact with thrombospondin and Tgfβ [26] and elevated *Dcn* expression reduces Tgfβ induced fibrosis in model systems [27]. Decorin deficiency results in a much more severe diabetic nephropathy in mice with streptozotocin induced diabetes [28]. It is interesting to note that Decorin is also significantly up-regulated in mesangial cells of mice with diabetic nephropathy [17].

The genes down regulated in the mutant mesangial cells also revealed interesting functional pathways (Additional file 6: Table S6). The most statistically significant altered biological process was sterol biosynthesis. Down regulated genes and encoded proteins included: *Fdft1*, encoding the first specific enzyme in cholesterol biosynthesis, catalyzing the dimerization of two molecules of farnesyl diphosphate to form squalene; Fdps catalyzes the production of farnesyl pyrophosphate,a key intermiedatein cholesterol and sterol biosynthesis; Sqle, drives the first oxygenation step in sterol biosynthesis and is thought to be a rate limiting enzyme; Dhcr7 catalyzes the conversion of 7-dehydrocholesterol to cholesterol; Idi1 synthesizes a precursor to farnesyl diphosphate; and Mvk, mevalonate kinase, an early enzyme in cholesterol biosynthesis. Also downregulated were genes involved in axogenesis, suggesting changes in cytoskeletal architecture. The muscle development gene expression signature was also reduced, as were genes associated with vasculature development, reflecting the changing character of the mesangial cells in the *Cd2ap* mutants.

Of interest, we observed robust expression of *Wt1* in mesangial cells, with RPKM values of 34 and 15 in control and mutant, respectively. This further confirms our previous report of *Wt1* expression in mesangial cells [17]. *Wt1* expression is generally considered a podocyte marker in the adult kidney. The observed *Wt1* expression in mesangial cells was not the result of podocyte contamination, as a number of other podocyte marker genes, including *Nphs1* (RPKM 3), *Nphs2* (RPKM 3), *Mafb* (RPKM 5.6) and *Sulf1* (RPKM 3) showed very low expression levels. The observed *Wt1* expression level in mesangial cells was nevertheless much lower (about 10 fold) than observed in podocytes, explaining the relatively specific podocyte expression previously reported using the poorly quantitative immunostain and *in situ* hybridization techniques.

### Podocytes

Although all three major cell types of the glomerulus contribute to the FSGS disease pathology it is generally agreed that perturbed podocyte function plays a central causative role. This is certainly true for *Cd2ap* mutants. We were therefore particularly interested in the perturbed gene expression profiles of the podocytes. We used the *MafB-GFP* transgene reporter, coupled with FACS, to isolate podocytes from wild type and mutant mice, as previously described [29].

**Fig. 3:**
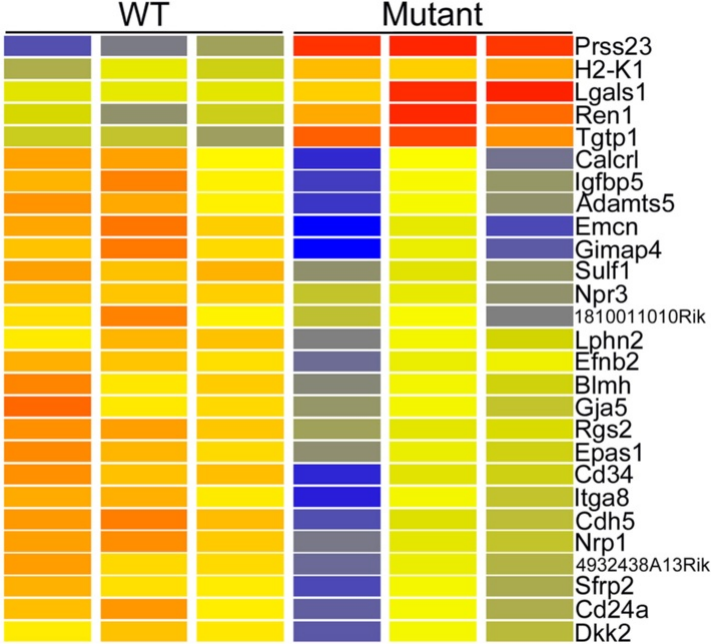
Heatmap of genes showing microarray based differential expression in *Cd2ap* ^−/−^ mutant podocytes. Genes were filtered for moderate to high expression (raw signal > 500) and Fold Change > 2. Red is high expression, blue is low expression and yellow is intermediate. Differential expression was validated for all of these genes by independent RNA-seq analysis

A relatively low stringency screen of the microarray data (P < 0.05, FC > 1.5) comparing wild type and mutant podocytes identified 73 annotated genes with higher expression in mutant podoctyes (Additional file 7: Table S7). Over 90 % (67 genes) were independently validated by RNA-seq. Increasing the stringency of the screen to require a robust raw expression level of 500, P < 0.05 and FC > 2 identified only five genes with elevated expression and 22 genes with reduced expression in mutant podocytes (Fig. 3).

The strongest up regulation was for *Prss23*, encoding a serine protease of the trypsin family. This is of particular interest because a number of studies have connected proteases to FSGS pathology [30–34]. *Prss23* is also up regulated in human FSGS patients, even when not caused by *Cd2ap* mutation, suggesting it may be a common feature of FSGS pathogenesis [35].

Of interest, our data did not show altered expression of another protease, cathepsin L, which has been previously strongly implicated in FSGS caused by *Cd2ap* mutation. Our array data showed high cathepsin L level expression (about 1500 raw signal) in both wild type and mutant podocytes and the RNA-seq data showed extremely strong expression of 432 RPKM in wild type and somewhat lower 325 RPKM for mutants. Again, this is of particular interest because a previous report concluded that mutation of *Cd2ap* results in an up-regulation of cathepsin L by causing the nuclear accumulation of the transcription factor dendrin [36]. The actions of cathepsin were then proposed to result in a reorganization of the podocyte microfilament system, resulting in proteinuria and increased apoptotic susceptibility to Tgfβ1 [36]. The apparent discrepancy between our data and the previous report suggested the possible involvement of another cathepsin, previously mistaken as cathepsin L, perhaps the result of imperfect antibody specificity. In looking at other cathepsin genes, however, most showed no change and a few showed modest changes, in both directions, but with very low expression levels compared to capthepsin L. In summary, contrary to a previous report, our microarray/RNA-seq data did not show up-regulation of cathepsin L in *Cd2ap* mutant podocytes.

Surprisingly, microarrays showed one of the up-regulated genes in mutant podocytes was renin (Fig. 3). This was also seen with RNA-seq, which showed a dramatic up-regulation of *Ren1* expression from 5 RPKM in wild type to 190 RPKM in mutant. The significance of this is uncertain, but it is interesting to note that cells of the renin lineage have been previously reported to serve as podocyte progenitors during glomerular disease [37]. Our expression data could therefore be interpreted as supporting such a renin expressing precursor-podocyte relationship.

As observed for the mesangial cells, the RNA-seq analysis (FC > 2) of the podocytes also identified more genes with stronger differential expression than seen by microarray, with 919 genes up-regulated (Additional file 8: Table S8) and 760 down-regulated (Additional file 9: Table S9) in mutants.

**Fig. 4:**
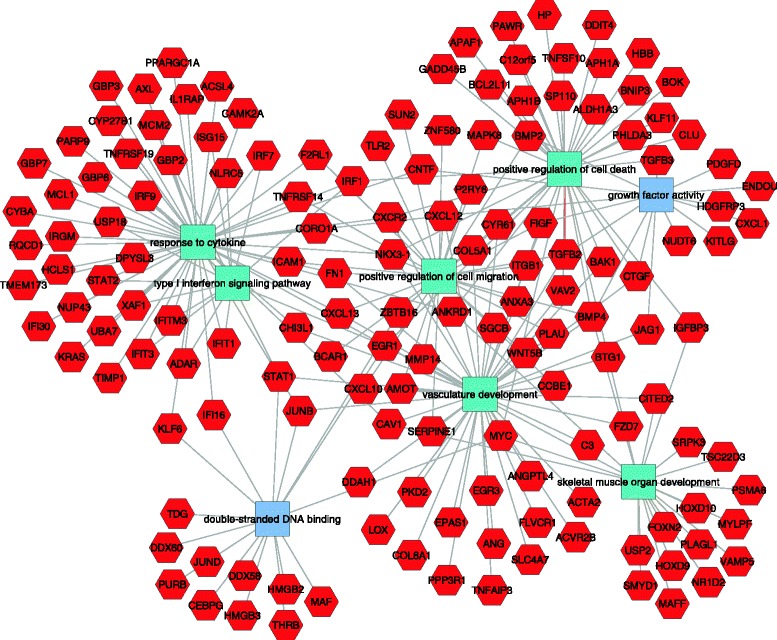
Functional analysis of genes upregulated in mutant podocytes. Genes are red hexagons and molecular functions and biological processes are rectangles. Differential expression was defined by RNA-seq. Key functional categories include positive regulation of cell death, vasculature development, muscle development and response to cytokines

Functional enrichment gene ontology analysis identified many interesting molecular functions and biological processes up-regulated in the mutant podocytes (Additional file 10: Table S10). Some of the most interesting were: response to cytokine (55 genes), extracellular matrix organization (32 genes), response to interferon (15 genes), muscle development (21 genes), vascular development (46 genes) and positive regulation of cell motility (28 genes) (Fig. 4). The mutant podocytes also showed a striking up-regulation of 49 genes associated with programmed cell death, including *Bak1* (Bcl2 antagonist/killer1), *Apaf1* (apoptotic peptidase activating factor 1), *Bok* (*Bcl2*-related ovarian killer protein), *Bcl2l11* (*Bcl2*-like 11 (apoptosis facilitator), *Moap1* (modulator of apoptosis 1), *Pawr* (PRKC, apoptosis, *Wt1*, regulator), *Pcdc2* (Programmed Cell Death 2), *Perp* (TP53 apoptosis effector) and many others, strongly suggesting an apoptotic response in the podocytes.

Other up-regulated genes of interest included six *Klf* transcription factor genes (*6,9,10,11,15,16*), fibronectin and five collagen genes (*12a1*, *5a1*, *5a3*, *6a1*, *8a1*). There was also a strong upregulation of *Tgfβ2*, *Tgfβ3*, tenascin c and nidogen.

A number of interesting genes were also down regulated in the mutant podocytes. Microarrays identified 311 annotated down regulated genes (P < 0.05, FC > 1.5), of which 75 % were validated by RNA-seq (Additional file 11: Table S11). RNA-seq, in turn, identified 760 down-regulated genes (Additional file 9: Table S9). Five keratin genes are expressed in podocytes and all were down regulated in the mutants. Keratins are fibrous proteins that provide strength and resilience to epithelial cells and their reduced expression likely reflects a dramatically altered character of the mutant podocytes. In addition several integrins were down regulated and *Myosin1d*, which showed very strong expression in wild type podocytes was reduced in expression in mutants by about three fold.

Several growth factors showed reduced expression in mutant podocytes. Pleiotrophin (*Ptn*) showed extremely strong expression in wild type (RPKM of 133) compared to mutant (RPKM of 33). Pleiotrophin has been implicated in driving neurite outgrowth, which is interesting considering the neurite like projections of the podocytes. *Egf*, *Bmp6* and *Nenf* (neuron derived neurotrophic factor) also showed reduced expression in mutants.

### Endothelial cells

**Fig. 5:**
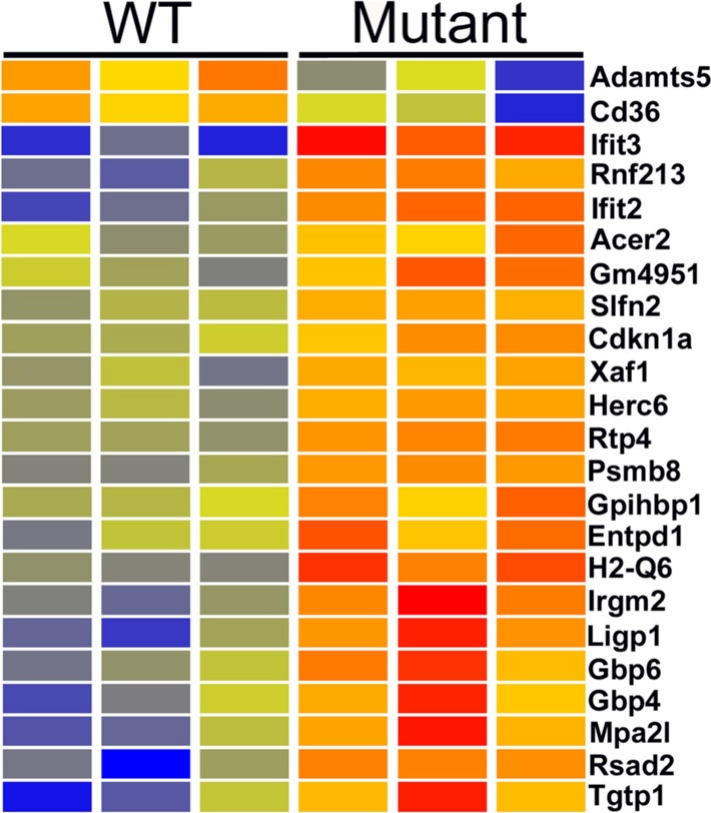
Heatmap of genes showing microarray based differential expression in *Cd2ap* ^−/−^ mutant endothelial cells. Genes were filtered for moderate to high expression (raw signal > 500) and Fold Change > 2. Red is high expression, blue is low expression and yellow is intermediate. Differential expression was validated for all of these genes by independent RNA-seq analysis

The third major cell type of the glomerulus, the endothelial cell, also plays a major role in FSGS disease progression, through the production of growth factors and cytokines, as well as the recruitment of macrophages and leukocytes. A low stringency microarray screen (P < 0.05, FC > 1.5) found 217 genes up-regulated in the *Cd2ap* mutant endothelial cells (Additional file 12: Table S12). All but five of the 211 annotated genes in this list were validated with the independent RNA-seq analysis. Microarrays identified a smaller number of down-regulated genes, 39, with all but four validated by RNA-seq (Additional file 13: Table S13). A more stringent screen of the microarray data (raw signal > 500, P < 0.05, FC > 2) identified 23 genes with the strongest microarray/RNA-seq cross-validated changes in expression (Fig. 5).

Once again RNA-seq found many more genes with altered expression. With a moderate stringency screen (RPMK > 3, P < 0.05, FC > 2), there were 355 up-regulated (Additional file 14: Table S14) and 348 down-regulated genes in the *Cd2ap* mutant endothelial cells (Additional file 15: Table S15). A ToppGene functional enrichment analysis found many of the up-regulated genes were related to response to cytokines and interferons (Additional file 16: Table S16). These included the interferon inducible *Ifit1*,*2*,*3 ifih1*, *ifi27, 35, 44, 47*,*204*, *Iigp1*, *Isg20*, *Stat1,2*, *Oas1a, 1b l2, H2-Abl* and the GTPases *Gbp2,3,4,5,6,7,8* and *9*.

The potent vasoconstrictor endothelin was strongly up-regulated, with its RPKM moving from 9 in wild type to 68 in mutant endothelial cells. In addition several growth factor encoding genes were strongly up-regulated, including *Bmp4* (RPKM 7.4 in WT and 38 in mutant), *Inhbb* (RPKM 0.03 WT and 3.0 mutant), *Mdk* (RPKM 2.2 WT and 6.6 mutant), *Pdgfa* (RPKM 8.5 WT and 17 mutant), *Pdgfb* (RPKM 38 WT and 75 mutant), *Cxcl10* (RPKM 2 WT and 6.6 mutant), *Cxcl12* (RPKM 1.1 WT and 5 mutant), *Igf2* (RPKM 5.5 WT and 19 mutant), *Ctgf* (RPKM 8 WT and 32 mutant) and *Il15* (RPKM 1.5 WT and 3.7 mutant).

**Fig. 6:**
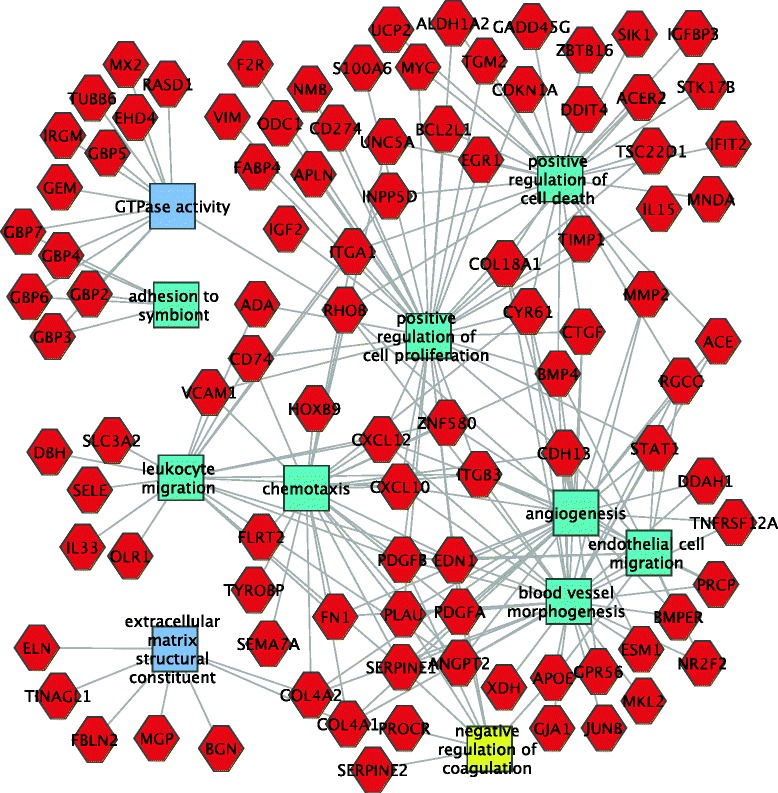
Functional analysis of genes upregulated in mutant endothelial cells. Genes are red hexagons and molecular functions and biological processes are rectangles. Differential expression was defined by RNA-seq. Key functional categories include positive regulation of cell death, positive regulation of cell proliferation, leukocyte migration, GTPase activity and chemotaxis

*Vcam1* and *Sele* were also up-regulated. These genes encode cell surface molecules that mediate adhesion of lymphocytes, monocytes, eosinophils and basophils to endothelium. Also of interest, fibronectin was up-regulated eight fold. A cytoscape showing some of the up-regulated genes and associated functions is shown in Fig. 6.

Functional enrichment analysis of genes down-regulated in mutant endothelial cells found, similar to that observed for mesangial cells, severe down-regulation of genes involved in sterol/cholesterol biosynthesis (Additional file 17: Table S17). It is also interesting to note that eight genes (*Nedd4l*, *Chrm3*, *Dmpk*, *Prkg*1, *Calca*, *Gstm2*, *Ppp1r12b*, *Pln*) involved in the regulation of muscle contraction were down-regulated.

### Additional validations

The primary validations for this study are in the form of dual global analysis of gene expression profiles with two distinct technologies, microarray and RNA-seq. We observed that typically over 90 % of genes called differently expressed with microarray, even with a low stringency screen of the data (FC > 1.5), were independently confirmed with RNA-seq. The reverse was not true, however, as many of the gene expression differences seen with RNA-seq were not detected by microarray. The double validated gene lists are therefore the most firm, while the much larger RNA-seq gene lists are the most inclusive.

**Fig. 7:**
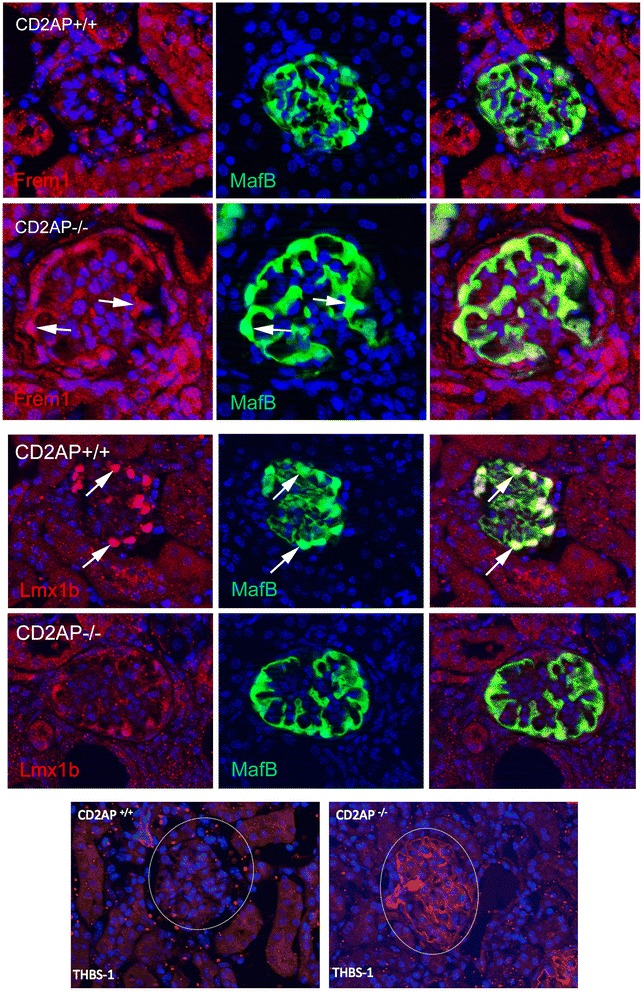
Immunostain validations of differential expression. Top panels show elevated expression of Frem1 in podocytes (MafB positive) cells of the glomerulus. Arrows show two cells with higher Frem1 levels in mutants. Middle panels show elevated expression of Lmx1b in wildtype podocytes. Arrows mark two cells with increased Lmx1b expression. Bottom panels show elevated expression of thrombospondin (Thsb1) in mutant mesangial cells

We selected a few genes to further validate, using immunostain to monitor levels of the encoded proteins. Expression for Frem1 was elevated, while Lmxb1 was reduced in mutant podocytes. Thsb1 showed higher expression levels in the mutant mesangial cells (Fig. 7).

## Conclusions

In this study we defined the changing properties of the three major cell types of the glomerulus in *Cd2ap*^−/−^ mutant mice. For each major cell type we observed both potentially pathogenic as well as protective shifts in gene expression. The mesangial cells showed dramatic upregulation of a number of key genes, including nephropontin, *Vegf*, endothelin, *Gdf6*, thrombospondin, *Tgfβ2* and *Tgfb3*. There was also strong upregulation of *Aldh1a2*, involved in retinoic acid synthesis and likely providing some disease protection. Similarly, the elevated expression of decorin, an antagonist of Tgfbeta, is probably protective. It was also interesting that the mesangial cells were observed to express significant levels of *Wt1*, with RPKM of about 30 in wild type. This is about one tenth of the very high expression level observed in podocytes, explaining why immunostains and *in situ* hybridizations generally define *Wt1* expression as podoctye specific.

In the podocytes we observed strong upregulation of the protease Prss23, but not the previously reported Cathepsin L [36]. Functional enrichment gene ontology analysis showed a number of pathways with strongly associated gene upregulation, including muscle development, vascular development, positive regulation of cell motility and programmed cell death. In addition all five keratin genes normally expressed in podocytes were strongly down-regulated, likely reflecting a dramatically altered podocyte character in the mutants. The growth factor pleiotrophin, which has been implicated in driving neurite outgrowth, also showed much lower expression in mutants.

The endothelial cells showed a number of interesting gene expression changes, including upregulation of the vasoconstrictor endothelin (also upregulated in mesangial cells), Bmp4, Midkine (which promotes angiogenesis) and many genes related to response to cytokines and interferons. *Vcam1* (vascular cell adhesion molecule 1) and *Sele*, which mediate leukocyte adhesion, were also upregulated.

In summary, this study provides a global RNA-seq analysis of the changing properties of the three major cell types of the glomerulus in the *Cd2ap* mutant mouse, identifying activated and repressed pathways and responsible genes. Because *Cd2ap* mutations have been associated with nephrotic syndrome and FSGS in humans [3, 5], the results of this study translate to a deeper molecular understanding of this genetic disease.

## Additional files

Additional file 1: Table S1. Microarray defined genes upregulated in *Cd2ap* mutant mesangial cells. Filtered for fold change > 1.5 and raw signal > 100. Columns show gene symbol, fold change, gene description, RNA-seq validation with P < 0.05, RNA seq validation fold change and transcript cluster ID. Additional file 2: Table S2. Microarray defined genes higher in wild type mesangial cells compared to *Cd2ap* mutant. Filtered for fold change > 1.5 and raw signal > 100. Columns show gene symbol, fold change, gene description, RNA-seq validation with P < 0.05, RNA seq validation fold change and transcript cluster ID. Additional file 3: Table S3. RNA-seq defined genes upregulated in *Cd2ap* mutant mesangial cells. Fold change > 2.0. Columns show gene symbol, fold change, Gene ID, RPKM in mutant, RPKM in wild type and gene position as determined by chromosome, start and end. Additional file 4: Table S4. RNA-seq defined genes higher in wild type mesangial cells compared to *Cd2ap* mutant. Fold change > 2.0. Columns show gene symbol, fold change, Gene ID, RPKM in mutant, RPKM in wild type and gene position as determined by chromosome, start and end. Additional file 5: Table S5. Functional enrichment analysis of genes with higher expression in *Cd2ap* mutant mesangial cells. Columns show functional category (molecular function or biological process), name of function, uncorrected P-value, FDR Benjamini and Hochberg corrected P value, Hit count in gene list, Hit count in genome, Genes with hit in gene list. Additional file 6: Table S6. Functional enrichment analysis of genes with higher expression in wild type mesangial cells. Columns show functional category (molecular function or biological process), name of function, uncorrected P-value, FDR Benjamini and Hochberg corrected P value, Hit count in gene list, Hit count in genome, Genes with hit in gene list. Note much lower P values compared to genes upregulated in mutant mesangial cells. Additional file 7: Table S7. Microarray defined genes upregulated in *Cd2ap* mutant podocytes cells. Filtered for fold change > 1.5 and raw signal > 100. Columns show gene symbol, fold change, gene description, RNA seq validation fold change and transcript cluster ID. Additional file 8: Table S8. RNA-seq defined genes upregulated in *Cd2ap* mutant podocytes. Fold change > 2.0. Columns show gene symbol, fold change, Gene ID, RPKM in mutant, RPKM in wild type and gene position as determined by chromosome, start and end, gene description. Additional file 9: Table S9. RNA-seq defined genes higher in wild type podocytes compared to *Cd2ap* mutant. Fold change > 2.0. Columns show gene symbol, fold change, Gene ID, RPKM in mutant, RPKM in wild type and gene position as determined by chromosome, start and end, gene description. Additional file 10: Table S10. Functional enrichment analysis of genes with higher expression in *Cd2ap* mutant podocytes. Columns show functional category (molecular function or biological process), name of function, uncorrected P-value, FDR Benjamini and Hochberg corrected P value, Hit count in gene list, Hit count in genome, Genes with hit in gene list. Additional file 11: Table S11. Microarray defined genes higher in wild type podocytes compared to *Cd2ap* mutant. Filtered for fold change > 1.5 and raw signal > 100. Columns show gene symbol, fold change, gene description, RNA seq validation fold change and transcript cluster ID. Additional file 12: Table S12. Microarray defined genes upregulated in *Cd2ap* mutant endothelial cells. Filtered for fold change > 1.5 and raw signal > 100. Columns show gene symbol, fold change, RNA seq validation fold change and transcript cluster ID. Additional file 13: Table S13. Microarray defined genes upregulated in *Cd2ap* wild type endothelial cells compared to *Cd2ap* mutants. Filtered for fold change > 1.5 and raw signal > 100. Columns show gene symbol, fold change, gene description, RNA seq validation fold change and transcript cluster ID. Additional file 14: Table S14. RNA-seq defined genes upregulated in *Cd2ap* mutant endothelial cells. Fold change > 2.0. Columns show gene symbol, fold change, Gene ID, RPKM in mutant, RPKM in wild type and gene position as determined by chromosome, start and end. And gene description. Additional file 15: Table S15. RNA-seq defined genes higher in wild type endothelial cells compared to *Cd2ap* mutant. Fold change > 2.0. Columns show gene symbol, fold change, Gene ID, RPKM in mutant, RPKM in wild type and gene position as determined by chromosome, start and end, gene description. Additional file 16: Table S16. Functional enrichment analysis of genes with higher expression in *Cd2ap* mutant endothelial cells. Columns show functional category (molecular function or biological process), name of function, uncorrected P-value, FDR Benjamini and Hochberg corrected P value, Hit count in gene list, Hit count in genome, Genes with hit in gene list. Additional file 17: Table S17. Functional enrichment analysis of genes with higher expression in wild type endothelial cells compared to *Cd2ap* mutant. Columns show functional category (molecular function or biological process), name of function, uncorrected *P*-value, FDR Benjamini and Hochberg corrected P value, Hit count in gene list, Hit count in genome, Genes with hit in gene list.

## Abbreviations

FSGS: Focal segmental glomerulosclerosis
FACS: Fluorescent activated cell sorting
GFP: Green fluorescent protein
RPKM: Reads per kilobase per million reads
WT: Wild type
